# Immunization with DNA Plasmids Coding for Crimean-Congo Hemorrhagic Fever Virus Capsid and Envelope Proteins and/or Virus-Like Particles Induces Protection and Survival in Challenged Mice

**DOI:** 10.1128/JVI.02076-16

**Published:** 2017-04-28

**Authors:** Jorma Hinkula, Stéphanie Devignot, Sara Åkerström, Helen Karlberg, Eva Wattrang, Sándor Bereczky, Mehrdad Mousavi-Jazi, Christian Risinger, Gunnel Lindegren, Caroline Vernersson, Janusz Paweska, Petrus Jansen van Vuren, Ola Blixt, Alejandro Brun, Friedemann Weber, Ali Mirazimi

**Affiliations:** a Department of Clinical and Experimental Medicine, Linköping University, Linköping, Sweden; b Microbiology and Tumorbiology Center, Karolinska Institutet, Solna, Sweden; c Institute for Virology, Philipps University Marburg, Marburg, Germany; d Institute for Virology, FB10, Justus Liebig University, Giessen, Germany; e Folkhälsomyndigheten, Stockholm, Sweden; f National Veterinary Institute, Uppsala, Sweden; g Department of Chemistry, University of Copenhagen, Frederiksberg, Denmark; h Centre for Emerging and Zoonotic Diseases, National Institute for Communicable Diseases, National Health Laboratory Service, Sandringham, South Africa; i Centro de Investigación en Sanidad Animal, Instituto Nacional de Investigación y Tecnología Agraria y Alimentaria, Madrid, Spain; j Department of Medicine, Karolinska Institutet, Stockholm, Sweden; Icahn School of Medicine at Mount Sinai

**Keywords:** Crimean-Congo hemorrhagic fever virus, DNA vaccines, Th1/Th2 responses, VLP, neutralizing antibodies

## Abstract

Crimean-Congo hemorrhagic fever virus (CCHFV) is a bunyavirus causing severe hemorrhagic fever disease in humans, with high mortality rates. The requirement of a high-containment laboratory and the lack of an animal model hampered the study of the immune response and protection of vaccine candidates. Using the recently developed interferon alpha receptor knockout (IFNAR^−/−^) mouse model, which replicates human disease, we investigated the immunogenicity and protection of two novel CCHFV vaccine candidates: a DNA vaccine encoding a ubiquitin-linked version of CCHFV Gc, Gn, and N and one using transcriptionally competent virus-like particles (tc-VLPs). In contrast to most studies that focus on neutralizing antibodies, we measured both humoral and cellular immune responses. We demonstrated a clear and 100% efficient preventive immunity against lethal CCHFV challenge with the DNA vaccine. Interestingly, there was no correlation with the neutralizing antibody titers alone, which were higher in the tc-VLP-vaccinated mice. However, the animals with a lower neutralizing titer, but a dominant cell-mediated Th1 response and a balanced Th2 response, resisted the CCHFV challenge. Moreover, we found that in challenged mice with a Th1 response (immunized by DNA/DNA and boosted by tc-VLPs), the immune response changed to Th2 at day 9 postchallenge. In addition, we were able to identify new linear B-cell epitope regions that are highly conserved between CCHFV strains. Altogether, our results suggest that a predominantly Th1-type immune response provides the most efficient protective immunity against CCHFV challenge. However, we cannot exclude the importance of the neutralizing antibodies as the surviving immunized mice exhibited substantial amounts of them.

**IMPORTANCE** Crimean-Congo hemorrhagic fever virus (CCHFV) is responsible for hemorrhagic diseases in humans, with a high mortality rate. There is no FDA-approved vaccine, and there are still gaps in our knowledge of the immune responses to infection. The recently developed mouse models mimic human CCHF disease and are useful to study the immunogenicity and the protection by vaccine candidates. Our study shows that mice vaccinated with a specific DNA vaccine were fully protected. Importantly, we show that neutralizing antibodies are not sufficient for protection against CCHFV challenge but that an extra Th1-specific cellular response is required. Moreover, we describe the identification of five conserved B-cell epitopes, of which only one was previously known, that could be of great importance for the development of diagnostics tools and the improvement of vaccine candidates.

## INTRODUCTION

Crimean-Congo hemorrhagic fever virus (CCHFV) is a member of the Nairovirus genus of the family Bunyaviridae. Viruses within this family possess three single-stranded RNA segments of negative sense. The large (L) segment encodes the RNA-dependent RNA polymerase, the medium (M) segment encodes the two structural glycoproteins (Gn and Gc) and a nonstructural protein (Nsm), and the small (S) segment encodes the nucleocapsid protein (N) and the nonstructural protein NSs (1). CCHFV is present in Asia, Africa, the Middle East, and Europe; in humans, it causes severe hemorrhagic disease with a reported mortality rate of up to 30%. CCHFV is transmitted through tick bites and/or through contact with viremic blood or tissues from patients or infected livestock (2).

The wide geographical distribution, mode of transmission, severity of the disease, and high mortality rate in humans, together with therapeutic difficulties and lack of an FDA-approved vaccine, make CCHFV a significant threat to public health.

Progress with CCHFV research has been severely hampered by the lack of a suitable animal model and by the requirement of high-containment laboratories to handle the virus. Newborn mice succumb to infection (3), but due to their immature immune systems, they cannot be used to assess vaccine efficacy. Recently, adult small-animal models have been developed using mice deficient in the antiviral type I interferon (IFN) signaling pathway, either in the type I IFN receptor (IFNAR) or in STAT1 (4, 5). CCHFV infection in IFNAR-knockout mice reproduces human disease via a variety of inoculation routes, including intradermal (i.d.)/subcutaneous injection, which is intended to mimic human infection by tick bite (6).

The only available vaccine is an inactivated virus produced on suckling mouse brain (inactivated by chloroform, heated at 58°C, and adsorbed on aluminum hydroxide). This vaccine is currently used in Bulgaria and originated from the Union of Soviet Socialist Republics in 1970 (7). Since 1974 it has been used to immunize mainly military and medical personnel, farmers, and persons living or working in regions of endemicity. A 4-fold reduction in the number of reported Crimean-Congo hemorrhagic fever cases in Bulgaria has been observed over a 21-year period following initiation of vaccination (8, 9). A recent study found that this vaccine elicited both a cellular and humoral response to CCHFV (9), but neutralizing antibody titers were low, even in individuals that who had received four doses (3, 7). Controlled studies on protective efficacy have not been reported with this vaccine, and, due to its crude preparation, it is unlikely to gain widespread international regulatory approval. There is, therefore, at present no safe and effective, commercially available vaccine against CCHFV.

Previously, Spik and colleagues (10) had demonstrated that a DNA-based vaccine expressing the CCHFV M segment induced neutralizing antibodies in approximately half of vaccinated mice; however, the lack of a challenge model at the time did not allow testing of protection. Another vaccine candidate using transgenic tobacco leaves expressing Gn and Gc was fed to mice and induced specific IgG and IgA. However, antibodies were not tested for virus neutralization (NT) capacity, and this oral vaccine could not be tested for protection either (11). Neither of these studies investigated cellular immune responses.

Recently, Buttigieg and colleagues (3) demonstrated that the attenuated poxvirus vector modified vaccinia virus Ankara expressing the CCHF virus glycoproteins (MVA-GP) raised cellular and humoral immune responses in two mouse strains, including IFNAR knockout mice, which are susceptible to CCHF disease. This vaccine protected all recipient animals from lethal disease in a challenge model adapted to represent infection via tick bite. Histopathology and viral load analysis of protected animals confirmed that they had been exposed to the challenge virus even though they did not exhibit clinical signs. This is the first experimental demonstration of efficacy by a CCHF vaccine.

Virus-like particles (VLPs) result from the self-assembly of recombinant viral proteins. Since they adopt a protein conformation and antigenicity similar to the native virus, VLPs are gaining more and more interest in the vaccine field, especially for emerging infectious disease viruses (12). Indeed, they can elicit strong humoral and cellular responses, and their lack of a viral genome renders them safer to use as vaccine than recombinant or attenuated/inactivated virus. We recently developed a transcriptionally competent VLP (tc-VLP) system for CCHFV (13) in which the particles display the two glycoproteins Gc and Gn at their surfaces and contain a reporter minigenome encapsidated by the nucleoprotein and bound by the viral polymerase. These tc-VLPs can be neutralized by specific CCHFV antiserum. For Rift Valley fever virus (RVFV), a bunyavirus of the Phlebovirus genus, tc-VLPs have been shown to induce innate immune responses and neutralizing antibody titers and to protect mice from lethal challenge without the need of an adjuvant (14, 15).

Here, we investigated the immunogenicity and protective efficacy of two novel vaccine candidates, a DNA vaccine coding for CCHFV Gc, Gn, or N and another using transcriptionally competent virus-like particles (tc-VLPs) (13), in the IFNAR^−/−^ mouse model and characterized the protecting humoral and cellular immune responses.

## RESULTS

### Antibody responses in immunized IFNAR^−/−^ mice.

**FIG 1:**
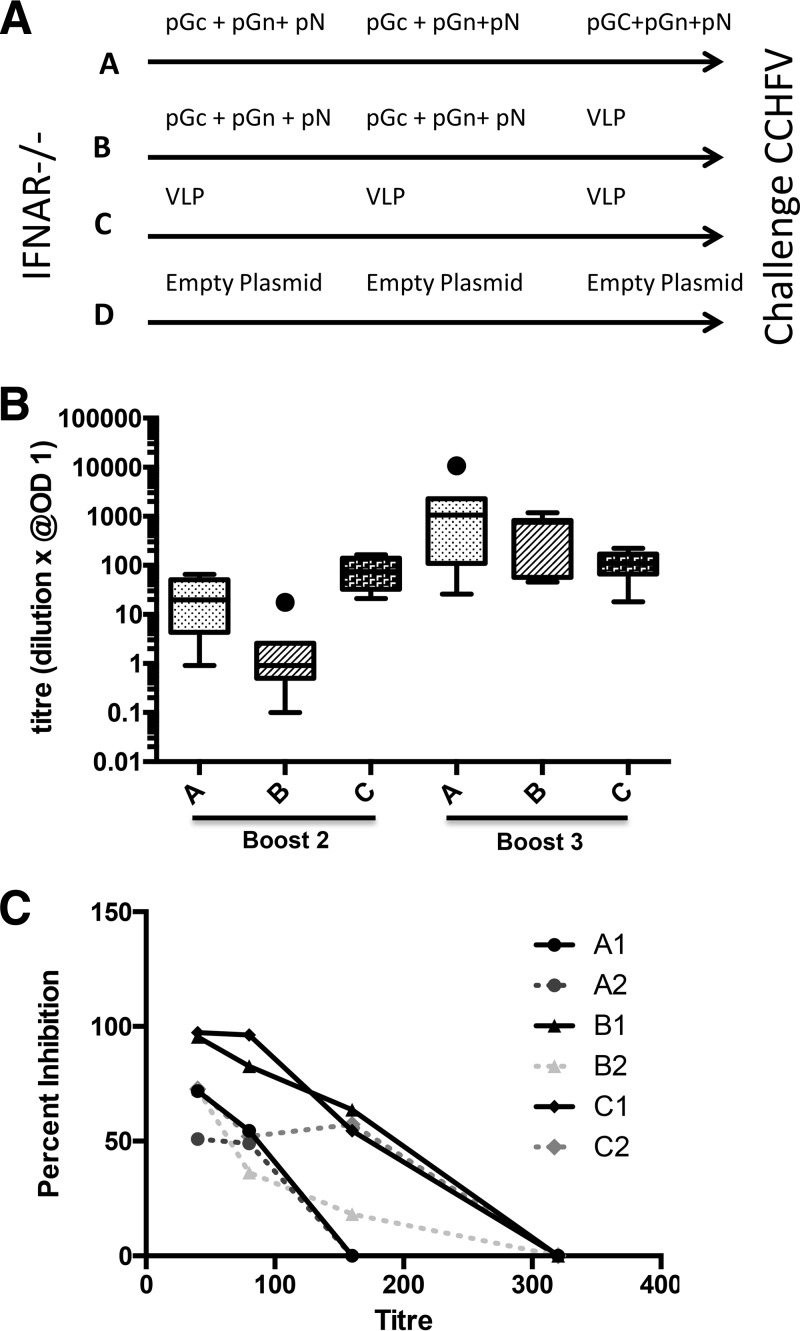
(A) A schematic drawing illustrating the immunization schedule for the A129 IFNAR^−/−^ mice used. Four groups of mice were compared: group A (three doses of CCHF-DNA-plasmids i.d. by Biojector administration), group B (two doses of CCHF-DNA-plasmids i.d. by Biojector administration and a final booster with CCHF-VLPs i.p.), group C (three doses of VLPs alone by i.p. injection), and group D (empty-DNA plasmids i.d. by Biojector administration). Immunizations were performed with 3-week intervals. Lethal CCHFV challenge was delivered by i.p. injection at 6 weeks post-final immunization. (B) Box plot representation of the total anti-CCHFV serum IgG titers after the second and third immunizations of the three groups of A129 IFNAR^−/−^ mice. The box encloses 50% of the data, with the median value indicated by a horizontal line; the limits of the box represent the upper and lower quartiles. Whiskers mark the maximum and minimum values, excluding outliers. Filled circles represent outliers, defined as values greater than the upper quartile or smaller than the lower quartile, +1.5× the interquartile distance. (C) Geometric mean of anti-CCHFV neutralizing serum titers/vaccine-receiving group. In each group the sera were pooled into two pools/subgroup based on the CCHFV ELISA IgG titers. In each subgroup the three mice with the highest serum IgG ELISA titers were pooled with equal volumes (serum pool A1, B1, and C1, respectively), and the mice with lowest serum IgG ELISA titers per group were pooled into pools indicated as A2, B2, and C2, respectively. OD, optical density.

The IFNAR^−/−^ mice were immunized with DNA plasmids encoding a ubiquitin (Ub)-linked version of CCHFV Gc, Gn, and N and/or with transcriptionally active virus-like particles (tc-VLPs). Ubiquitin-linked antigens may promote cellular responses more efficiently than unmodified antigens, thus broadening the immune response elicited. This has been shown for Rift Valley fever virus, another bunyaviru*s*, to enhance levels of specific antibodies and to induce a high degree of protection from challenge (16). The immunization schedules for IFNAR^−/−^ mice with the CCHFV antigens are depicted in Fig. 1A. The serum IgG responses to the CCHFV antigens were analyzed by enzyme-linked immunosorbent assay (ELISA). As shown in Fig. 1B, mice that were immunized with tc-VLPs alone reached the highest serum titers after two immunizations. However, after the second booster, antibody levels to CCHFV antigen increased dramatically for groups A and B in contrast to levels of the group which received only tc-VLPs (Fig. 1B).

To study neutralization (NT) specificity of antibodies raised by the DNA plasmids and/or tc-VLPs, we divided the mice in each group in two subgroups: those with the highest anti-CCHFV serum IgG antibody responses (subgroups A1, B1, and C1, with sera from three or four mice each) and those with the lowest anti-CCHFV serum IgG antibody responses (subgroups A2, B2, and C2, with sera from three mice each). The sera were pooled in each subgroup and were used for future studies. All immunized mouse groups responded by developing neutralizing antibodies (Fig. 1C). However, the mice that had been immunized exclusively with tc-VLPs (group C) consistently displayed higher neutralizing antibody titers than mice that received only DNA. Group B that received DNA twice and tc-VLPs once showed higher neutralizing antibody titers than mice that received only DNA.

### Survival by Kaplan-Meier analysis.

**FIG 2:**
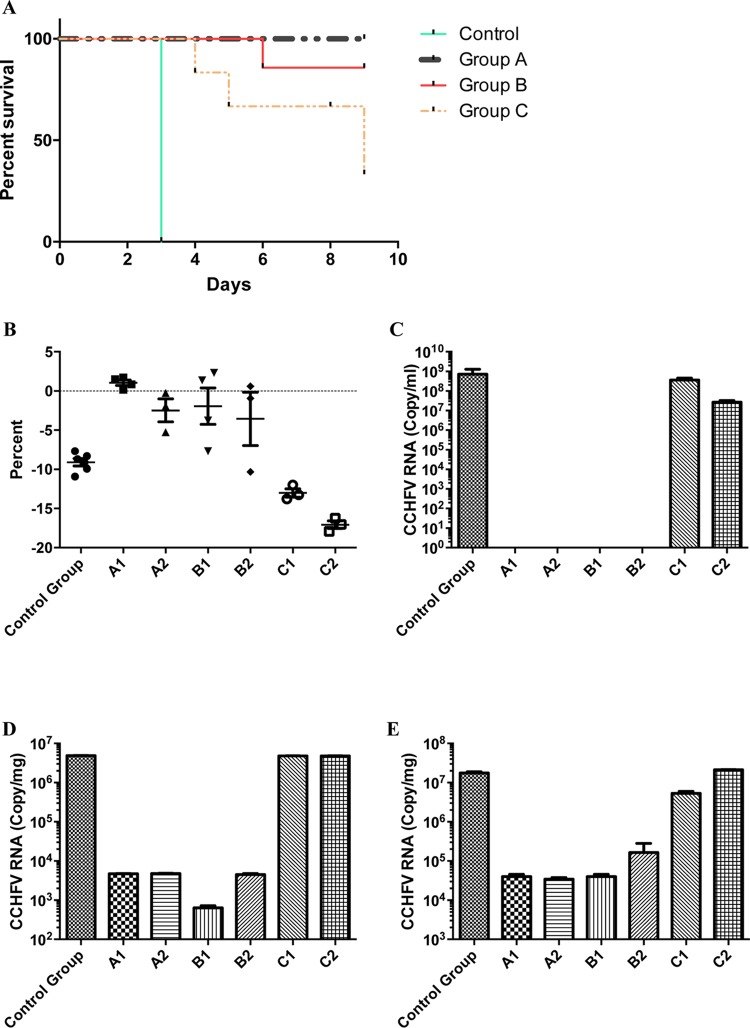
(A) Kaplan-Meier graph showing the survival pattern for each group of vaccinated and CCHFV-challenged A129 IFNAR^−/−^ mice. (B) The mean percentage of body weight change/group is shown post-CCHFV challenge (mean and 95% standard deviation shown for each group). Group A1 data represent animals with highest neutralizing serum antibody titers, and group A2 data represent two mice with the lowest serum neutralizing titers. Group B1 data show body weight changes in animals with highest neutralizing serum antibody titers, and group B2 data represent the two mice with the lowest serum neutralizing titers. Group C data represent mice immunized with three doses of tc-VLPs before CCHFV challenge. Group C1 data show body weight changes in animals with highest neutralizing serum antibody titers, and group C2 data represent two mice with the lowest serum neutralizing titers. (C to E) Quantitative mean CCHF viral RNA levels in serum (C), spleen (D), and liver (E) per group, as indicated.

Mouse survival was monitored for 10 days after lethal challenge with CCHFV. All mice in group A and all mice except one in group B survived the whole observation period after challenge. The full comparison of survival between immunized mouse groups and non-CCHFV-immunized mice is shown in Fig. 2A. The body weight of all mice at sacrifice (or at day of death due to CCHFV infection) is summarized in Fig. 2B. All A129 IFNAR^−/−^ mice receiving only CCHFV DNA plasmids (group A) survived without significant body weight loss (Fig. 2B). Group D (immunized by empty plasmids) animals all succumbed within 3 days, and the two actively immunized groups of mice survived at rates of 100% in the DNA plasmid-only group (group A) and of 80% in the DNA and tc-VLP group (group B). Surprisingly, in mice given only tc-VLPs, we found only a 40% survival rate and a significant body weight loss. Viral RNA load was analyzed by reverse transcription-PCR (RT-PCR) in blood, spleen, and liver (Fig. 2C to E) at the day of sacrifice. In control group D (nonimmunized) and in mice immunized with tc-VLPs (group C), we found high levels of viral RNA in all tested tissues. In contrast, in groups A and B, we could clearly demonstrate that mice cleared the virus from their blood as there was no viral RNA detectable in their sera (Fig. 2C). However, there was detectable viral RNA in the spleen and liver of the survivors (groups A and B). The level of viral RNA in spleen and liver in groups A and B was 10^3^ to 10^4^ times less than that in control group D and in group C. To investigate whether infectious virus was present at the end of the study in these organs, homogenized supernatants of spleen and liver tissue from survivors (groups A and B) and nonimmunized or tc-VLP-immunized mice were incubated with Vero cells for 72 h. Cells were fixed at 72 h postinfection (hpi) and analyzed by immunofluorescence assay (IFA) for the presence of CCHFV N protein. In contrast to our findings for control group D and the tc-VLP-immunized group C, we could not detect any infected cells in the cell culture infected with homogenate organs from groups A and B (data not shown). These results suggest that the small amounts of RNA detected in spleen and liver from groups A and B are unlikely to represent infectious virus particles. However, it is possible that the number of infectious virus particles in these homogenized organs was too low for detection with IFA.

### Cytokine response in IFNAR^−/−^ mice before the challenge.

**FIG 3:**
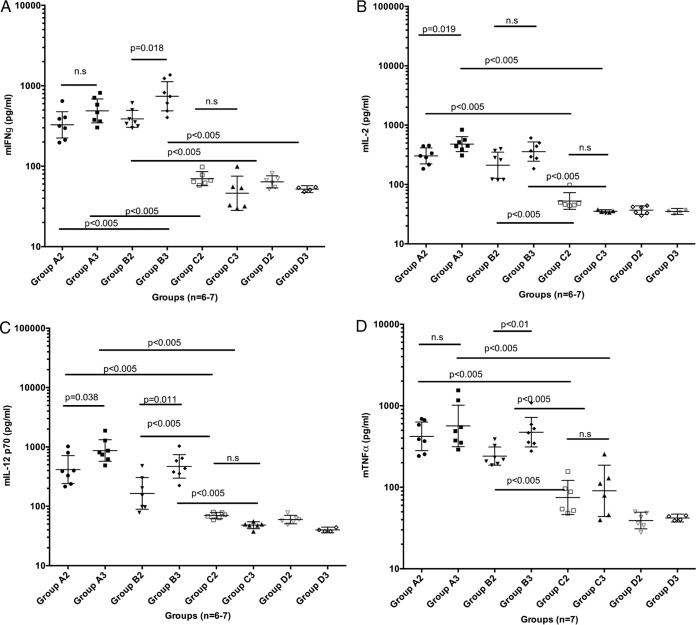

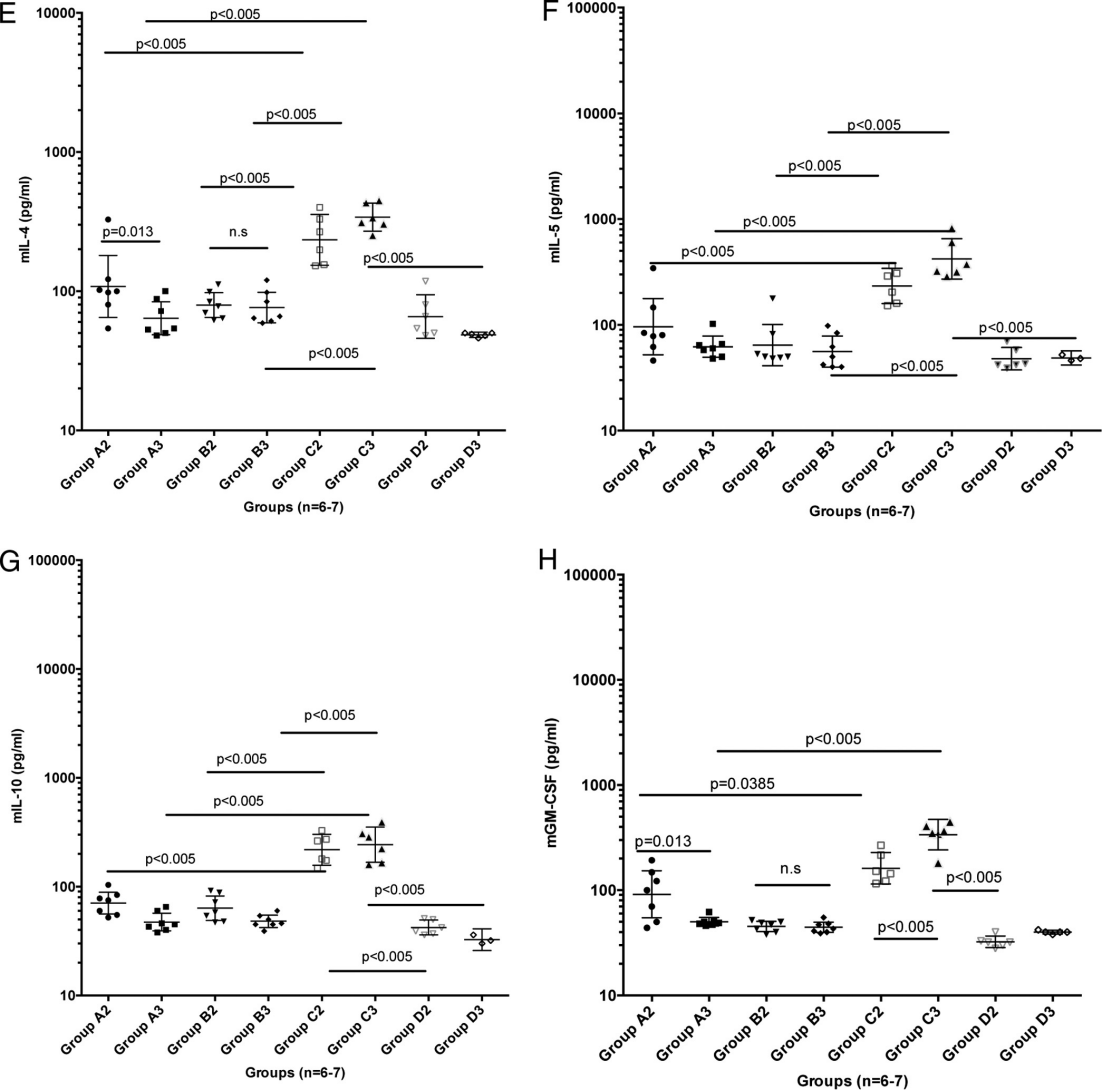
(A to D) Th1 cytokine determination in serum samples collected after two (A2, B2, C2) and three (A3, B3, C3) immunizations before challenge, from the four groups (A, B, C, and D). Each of the four panels illustrates individual mouse serum levels and group geometric mean levels at 95% confidence intervals of IFN-γ, IL-2, IL-12p70, and TNF-α, respectively. (E to H) Th2 cytokine determination in serum samples collected after two (A2, B2, C2) and three (A3, B3, C3) immunizations but before challenge, from the four groups (A, B, C, and D). Each of the four panels illustrates individual mouse serum levels and group geometric mean levels at 95% confidence intervals of IL-4, IL-5, IL-10, and GM-CSF, respectively. A Mann-Whitney test was made for group-wise comparison, and a *P* value of <0.05 was considered significant. n.s., not significant.

The prechallenge sera from the mice in groups A to D were monitored for their Th1 (Fig. 3A to D) and Th2 (Fig. 3E to H) cytokine profiles. The animals in group A and group B showed levels of the Th1 cytokines IFN-γ, interleukin-2 (IL-2), IL-12p70, and tumor necrosis factor alpha (TNF-α) that were significantly higher than those recorded for group C and the negative-control animals (Fig. 3A to D). In contrast, in group C levels of IL-4, IL-5, IL-10, and granulocyte-macrophage colony-stimulating factor (GM-CSF) were significantly higher than those recorded for groups A and B and the negative-control animals (Fig. 3E to H). This suggests the induction of a Th1-type immunity in DNA-immunized mice and of a Th2-type immunity in tc-VLP-immunized mice.

### Cytokine response of IFNAR^−/−^ mice after the challenge.

The cytokine profile for Th1 and Th2 responses was also investigated postchallenge in the surviving mice of groups A and B, whereas very small amounts of available sera prevented us from performing cytokine analyses for groups C and D.

**FIG 4:**
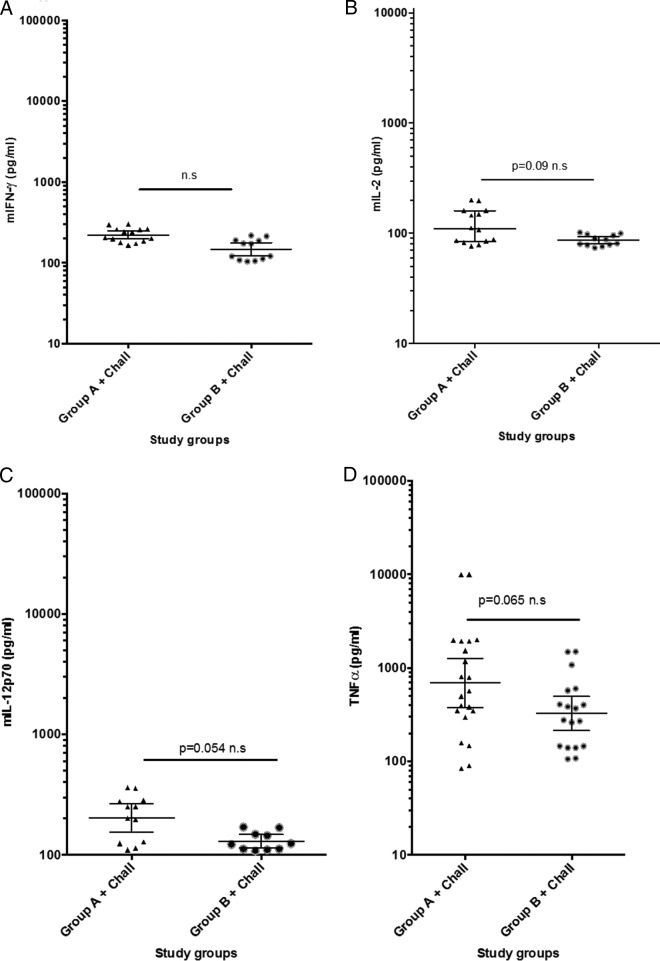

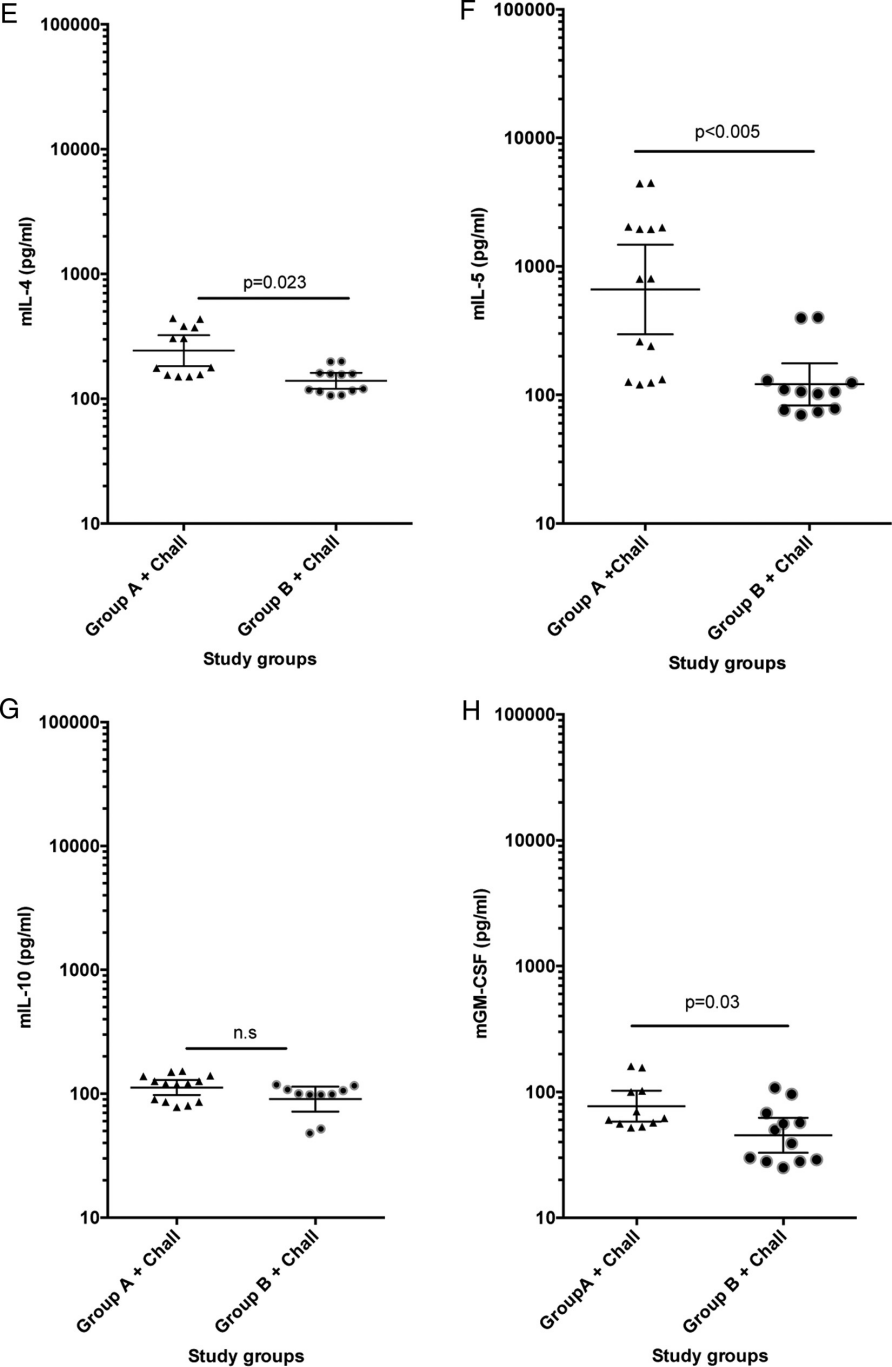
(A to D) Cytokine determination in serum samples collected from two groups, A and B, of immunized mice postchallenge. An analysis is shown of levels of Th1-pattern cytokines prior to experimental CCHFV challenge. Each of the four panels illustrates individual mouse serum levels in duplicate and group geometric mean levels at 95% confidence intervals of IFN-γ, IL-2, IL-12p70, and TNF-α, respectively. (E to H) Cytokine determination in serum samples collected from two groups, A and B, of immunized mice postchallenge. An analysis is shown of levels of Th2-pattern cytokines postchallenge. Each of the four panels illustrates individual mouse serum levels in duplicate and group geometric mean levels at 95% confidence intervals of IL-4, IL-5, IL-10, and GM-CSF, respectively. A Mann-Whitney test was made for group-wise comparison, and a *P* value of <0.05 was considered significant.

In both groups A and B the levels of IFN-γ, IL-2, IL-12p70, and TNF-α were significantly higher than those in the negative-control sera that were collected prior to immunizations (*P* = −0.001), again indicating a Th1-type immunity in the DNA-immunized mice (Fig. 4A to D).

In addition, we found that both group A and group B (Fig. 4E to H) showed significantly higher levels of IL-4 than the control group (*P* = 0.007 to 0.011), while only group A showed significantly higher levels of IL-5 and GM-CSF than the control group of nonimmunized or challenged mice.

**FIG 5:**
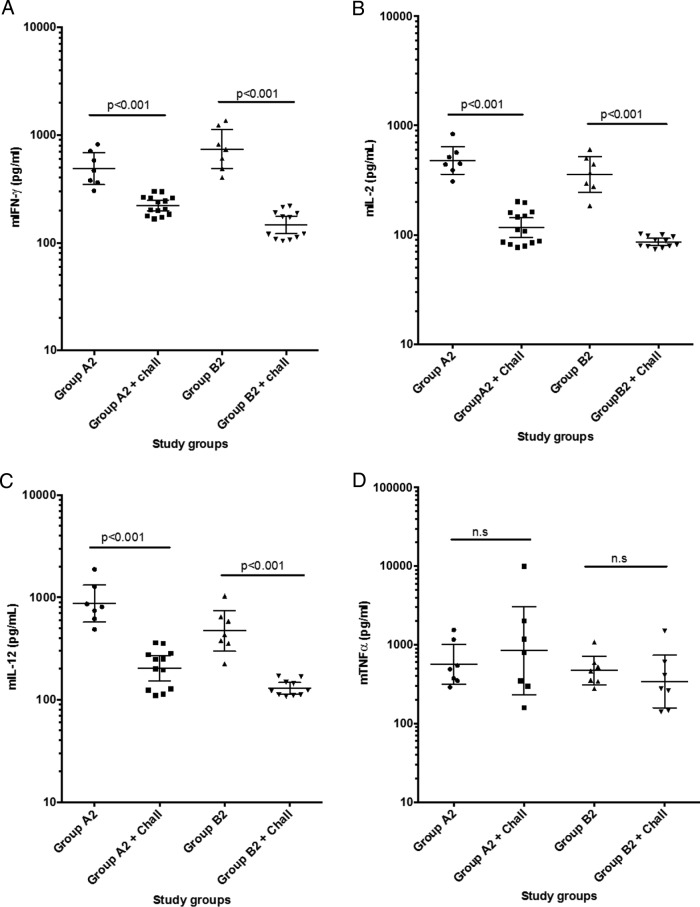
Comparison of cytokine patterns prior to and postchallenge in groups A and B. In panels A to D, the cytokine patterns of four Th1-type biomarkers are compared pre- and postchallenge; data are geometric means (95% confidence intervals). In panels E to H, the cytokine patterns of four Th2-type biomarkers are compared pre- and postchallenge; data are geometric means (95% confidence intervals).

Most interestingly, we found that the Th1 cytokine responses in groups A and B were shifted toward Th2 cytokine responses at 9 days postchallenge (Fig. 5A to H).

### IgG subclass determination.

**FIG 6:**
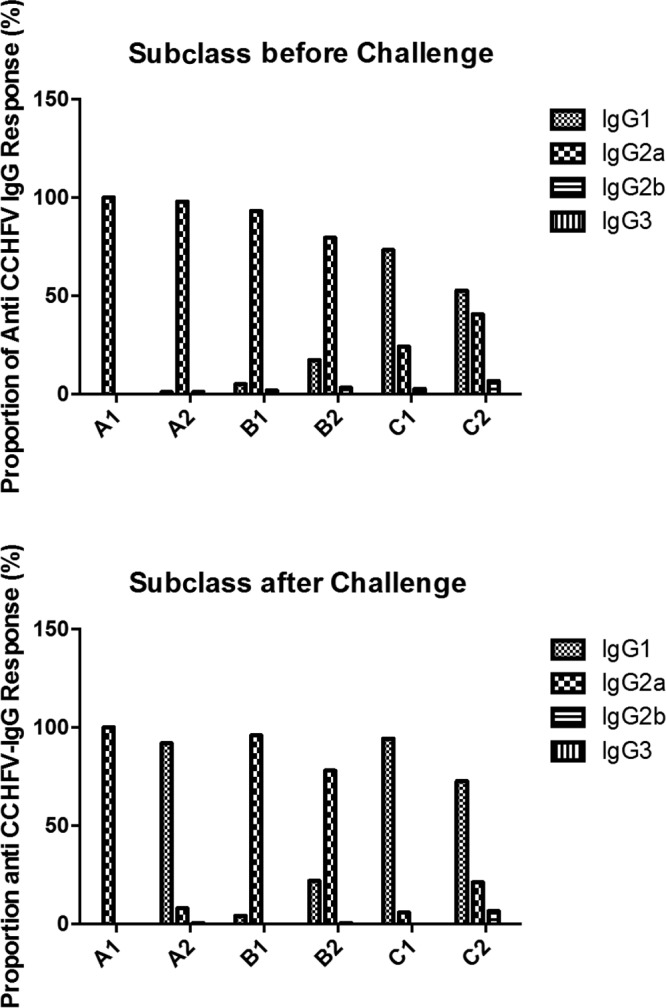
Serum IgG subclass reactivity for each pooled A129 IFNAR^−/−^ serum sample to CCHFV antigen. In the top panel, the subclass pattern of the serum pools (indicated on the *x* axis) is shown prior to CCHFV challenge, and in the bottom panel the serum IgG subclass pattern is shown at 10 days post-CCHFV challenge (at day of sacrifice).

We determined the subclasses of IgG antibodies to CCHFV as an indicator of Th1 and Th2 responses. For all groups of immunized mice, measurements were again separately performed for pooled sera with high total IgG responses (A1, B1, and C1) and pooled sera with low total IgG responses (A2, B2, and C2). All immunized mice clearly responded with IgG1, IgG2a, and IgG2b but not with IgG3, albeit the IgG2b responses constituted a small proportion of the total response in all groups (Fig. 6). The dominating subclasses were IgG1 and IgG2a, but response profiles varied between groups. All mice in group A (mice receiving only DNA plasmids) showed a higher IgG2a response than IgG1 response before the challenge, again indicating a Th1-type immune profile. Interestingly, the profile for group A2 was reversed after the challenge. In group B (two DNA plasmid immunizations and one tc-VLP immunization), all mice responded with a predominantly IgG2a serum response; however, levels in these mice indicated slightly higher IgG1 responses than in group A. It should be noted that the data from cytokine profiling (Fig. 5) suggest that groups A1 and -2 and groups B1 and -2 shift from Th1 to Th2 responses.

The third group of mice (C) (three doses of CCHFV tc-VLPs) had a clearly greater IgG1 than IgG2a response; however, group C1 had a predominantly IgG1 response in contrast to the C2 group, which demonstrated a relatively balanced IgG1 and IgG2a response before challenge. The proportion of IgG1 clearly dominated IgG2a in all mice in group C after challenge.

Altogether, we find that a clear difference in the Th1/Th2-type profiles evolved in the two types of immunization schedules, with DNA plasmid immunizations resulting in a predominantly Th1-type immunity and the VLP-based immunized animals demonstrating a predominantly Th2-type immune response.

### B-cell epitopes.

Seven linear B-cell epitopes in the CCHFV envelope proteins could be identified by screening of a synthetic peptide library with serum samples collected after challenge with CCHFV. The most frequent serum IgG responses (9/10 tested serum samples) were seen with a peptide representing the N-terminal Gn protein: CSIGSVNGFEIESHKCYCSL/IESHKCYCSLFCCPYCRHCS. The second most common serum IgG responses (6/10) were against the Gc epitope RLTSDGLARHVTQCPKRKEK/VTQCPKRKEKVEETELYLNL, 5/10 sera reacted with the mucin-like antigen epitopes TINCINLRASVFKEHREIEI and KIGKASTGFRKLLSEEPGDD, and 6/10 sera reacted to the Gc epitope LKGDRVNGHLIHKIESHFNT (Table 1). Interestingly, reactivity toward the mucin-like domain (MLD) epitopes could be predominantly seen in sera from mice of group B (4/5 tested serum samples), whereas only one individual serum sample from group A (1/5 tested serum samples) seemed to show an antibody response. Alignment of the identified B-cell epitopes in the Gn and Gc regions give an indication that they could be considered to represent pan-strain epitopes (data not shown). This finding could be of high interest for the development of diagnostic tools as well as new vaccine strategies.

**TABLE 1:** Reactive B-cell epitopes

Epitope (peptide)	Protein	No. of mice positive for the epitope	
		Group A (*n* = 5)	Group B (*n* = 5)
TINCINLRASVFKEHREIEI	Mucin-like	1	4
KIGKASTGFRKLLSEEPGDD	Mucin-like	1	4
RLTSDGLARHVTQCPKRKEK	Gn	3	3
––––––––––VTQCPKRKEKVEETELYLNL		2	3
CSIGSVNGFEIESHKCYCSL	Gn	3	2
––––––––––IESHKCYCSLFCCPYCRHCS		4	3
LKGDRVNGHLIHKIESHFNT	Gc	3	3

## DISCUSSION

The development of a vaccine for CCHFV has been severely hampered due to the lack of a suitable animal model and the requirement of a high-containment laboratory to handle the virus. We along with others recently developed adult small-animal models (mice deficient in the type I IFN signaling pathway), which replicate human disease via a variety of inoculation routes, as good models for development of vaccines and antivirals (5, 6).

In the current study, we used DNA plasmids expressing a ubiquitin-linked version of Gc, Gn, and N and/or tc-VLPs to immunize IFNAR^−/−^ mice. The immunized mice were challenged with CCHFV, and the immune responses before and after infection were studied. In this work, we demonstrated a clear and 100% efficient protective immunity against lethal CCHFV challenge if DNA plasmids expressing CCHFV proteins Gc, Gn, and N were given three times epidermally/intradermally to CCHFV-susceptible A129 IFNAR^−/−^ mice. We also found that a combination of DNA and VLP immunization can protect against the disease.

In contrast to most other CCHFV vaccine studies, the present study investigated the systemic pattern of eight selected Th1 and Th2 cytokines prior to and after the challenge. We have investigated the levels of Th1-type biomarkers IFN-γ, TNF-α, IL-12 p70, and IL-2, which were shown to be significantly elevated in the DNA-immunized and in the DNA- and tc-VLP-immunized A129 IFNAR^−/−^ mice prior to challenge. This may explain why these vaccinated animals were better prepared to respond to virus challenge before a systemic infection could occur, and thereby full protection was obtained. In addition, we observed a significantly higher IFN-γ response in group A (three DNA immunizations) than in group B (two DNA immunizations plus one tc-VLP immunization), as well as a tendency toward higher IL-2, IL-12p70, and TNF-α levels postchallenge. Perhaps these slight differences between the two protected groups, together with the IgG subclass pattern (Fig. 6) prior to challenge, suggest the level and breath of Th1 proinflammatory responses necessary to find a correlate of protection to the adaptive cell-mediated immune response pattern. Interestingly, the mice immunized with Ub-DNA plasmids did not demonstrate a clear Th2 response prior to the challenge. This result was not completely unexpected since Th1-type immune responses are often seen with DNA plasmid vaccines and especially with DNA constructs promoting ubiquitin pathway antigen processing (17). A comparison between the cytokine profiles before and after challenge demonstrated that there is a shift from a Th1 to Th2 response in groups A and B (Fig. 5A to H). However, the data from IgG subclass profiling demonstrate that only group A2 shifted to Th2. The difference between the outcomes of both cytokine and IgG subclass profiling could arise because cytokine detection is much more sensitive than IgG subclass detection and/or because the shift in IgG subclasses occurs later than 9 days postchallenge. However, we were able to test the serum IgG subclass patterns before and after challenge. These results confirm the data from the cytokine analyses. Thus, in this study the animals with the most elevated cell-mediated Th1 response combined with a good humoral Th2 type immune response resisted weight loss and lethal challenge. All CCHFV antigen-immunized mice developed detectable levels of neutralizing serum antibodies. However, animals with high neutralizing antibody titers and a Th2-type immune profile (tc-VLPs; group C) were only poorly protected. Thus, one may suggest that the protective immunity correlated strongly with a dominant Th1-type immune profile with a balanced Th2-type biomarker response, and, as determined by serum IgG subclass analysis, a strong IgG2a response was detected in the best-protected animals. Furthermore, in the two challenged and surviving groups (groups A and B), we could identify linear IgG B-cell epitopes to the CCHFV envelope proteins. In 10 of 12 tested serum samples (5 each in groups A and B) it was possible to identify responses to B-cell epitopes. As far as we know, only one of these epitopes has been described in CCHFV-immunized animals previously (18, 19). In two of the protected animals, serum IgG reactivity against linear CCHFV peptide B-cell epitopes could not be shown.

Monoclonal antibodies against the Gn envelope protein have been described as being more efficient in protecting mice against lethal CCHFV challenge than monoclonal antibodies to the Gc envelope protein (20). However, the Gc protein is considered more immunogenic (21). The majority of B-cell epitopes are in general directed to discontinuous epitopes that may be difficult to identify with short linear peptides (22). Therefore, it was interesting to see that this study could identify up to five different B-cell epitope regions (Table 1), representing both the envelope proteins Gn and Gc. Whether any of these epitopes are targets for virus-neutralizing antibodies remains unclear and requires further exploration.

Recently, Buttigieg and colleagues (3) demonstrated that vaccination with the attenuated poxvirus vector modified vaccinia virus Ankara expressing CCHFV glycoproteins (MVA-GP) can protect IFNAR^−/−^ mice against CCHFV challenge. The MVA-GP vaccine induced comprehensive cellular and humoral immunity to CCHFV. The protection was suggested to be correlated with T-cell responses but not with neutralizing antibody induction. Moreover, Canagloku and colleagues demonstrated that immunization with the cell culture-based inactivated vaccine elicited 80% protection against challenge with CCHFV in IFNAR^−/−^ mice (23). These authors suggested that a strong neutralizing antibody response is essential for the increased protection of IFNAR^−/−^ mice; however, the level of the T-cell-mediated immune response in mice was not measured. Also in another study using Gn and Gc subunit recombinant proteins with adjuvant, high titers of *in vitro* neutralizing antibodies were obtained in STAT1 knockout A129 mice (21). However, no protection was seen in the vaccinated animals, confirming that serum neutralizing antibodies alone are not protective in mice innately deficient in IFN type I/II. Thus, it seems likely that a broader immunity is required, including a cell-mediated immunity capable of clearing infected cells and suppressing virus replication, as well as antibodies capable of virus neutralization, antibody-dependent cellular cytotoxicity (ADCC), and complement-mediated viral lysis (24). These protective correlates are totally different from what has been described from Rift Valley fever (RVF) vaccine responses and passive immunotherapy with neutralizing antibodies followed by challenge in mice. In RVF virus infection, neutralizing antibodies correlate well with protection from infection and pathogenesis in mice (10). One possible explanation for these differences may be similar to what has been described in repeated dengue fever virus infections, namely, that the developed antibodies may contain both virus-neutralizing and *in vivo* infection-enhancing antibody populations and that the balance between these types of serum antibodies may influence the outcome of the infection.

The viral glycoproteins are often a first choice as antigens because they are expressed on the particle surface and can elicit neutralizing antibodies. However, there is strong sequence variation in the glycoprotein amino acids between CCHFV strains (25). It has been reported that the nucleoproteins of bunyaviruses, more conserved at the amino acid level, are also immunogenic and can confer partial protection after challenge, as shown for Rift Valley fever virus (14, 16, 26). In our study, we included the nucleoprotein N in the DNA immunization. Moreover, the nucleoprotein is also present in a more natural structure in the tc-VLPs. While we did not find any B-cell epitope within this protein, the nucleoprotein could be internalized and processed into antigen-presenting cells and contribute to T cell activation.

Interestingly, while the tc-VLPs for RVFV have been described to induce high neutralizing antibody titers and protection of mice upon challenge (5, 14), we did not observe such a correlation in the CCHFV system. The immunization with CCHFV tc-VLPs conferred a high neutralizing antibody titer, but only 40% of the mice survived the CCHFV infection. However, it is important to notice that the mice used in the RVFV study were wild-type and IFN-competent mice, while the ones used in our study are IFN incompetent. Indeed, wild-type mice do not show clinical signs of CCHFV infection, and so far only the IFNAR^−/−^ and STAT1^−/−^ mice have been described to develop disease (4, 5).

The tc-VLPs contain a ribonucleoprotein (RNP) complex consisting of a reporter gene encapsidated by the nucleoprotein and bound by the viral RNA polymerase (13). It has been demonstrated that incoming RNP complexes from several viral families, including RNPs from RVFV tc-VLPs, are sufficient to activate RIG-I and lead to IFN production (15). Moreover, IFN production is a good predictor of vaccine efficiency. tc-VLPs could induce better protection in an IFN-competent system, but, unfortunately, the IFN response and its implication in immunization by tc-VLPs cannot be assessed in our IFNAR^−/−^ mouse system.

In humans, CCHFV infection has been described to be divided into four phases, the incubation of 1 to 13 days, the prehemorrhagic phase of 3 days up to 1 week, the hemorrhagic phase of 2 or 3 days, and the final convalescence period approximately 2 weeks postsymptoms (27, 28). In the mouse IFNAR^−/−^ model, the process is considerably faster and more dramatic. This could explain why much of the specific immune response needs to be rapid, alert, and active to provide protection.

In a human clinical trial where the inactivated Bulgarian CCHFV vaccine was tested, mainly a cell-mediated immune response was shown (7). Up to four immunizations were needed to obtain detectable neutralizing serum antibody titers.

In conclusion, it seems that a predominantly Th1-type immune response, combined with mainly neutralizing antibodies, provides the most efficient protective immunity against CCHFV challenge given intraperitoneally (i.p.) in IFNAR^−/−^ mice.

## MATERIALS AND METHODS

### Animals.

Female, 6- to 8-week-old A129 IFNAR^−/−^ mice (The Jackson Laboratory, Sulzfeld, Germany) were used. Mice were housed according to the Karolinska Institute ethical rules, and all experimental protocols were approved by the regional Ethical Committee for animal research.

### Virus.

The CCHFV strain (Nigerian IbAr10200; the number of passages of this virus is not known) (5, 29) was serially diluted 10-fold and then titrated on Vero-E6 cells in 96-well plates. At 24 h postinfection, cells were fixed with 80% acetone and stained by immunofluorescence. The number of fluorescent foci in each well was counted, and the titer was determined using a rabbit polyclonal anti-CCHFV nucleocapsid antibody, diluted in phosphate-buffered saline (PBS) containing 0.2% bovine serum albumin (BSA) and 0.1% Triton X-100, followed by fluorescein isothiocyanate (FITC)-conjugated anti-rabbit antibody, as described previously (30).

### Plasmids.

The plasmids for production of tc-VLPs, pCAGGS_V5_L_wt (encoding the polymerase), pCAGGS_N (encoding the nucleoprotein), pT7riboSM2_vL_Ren (encoding the CCHFV-specific Renilla [REN] minigenome), pCAGGS_GP (encoding the full open reading frame [ORF] of the M segment), pCAGGS_T7 (encoding the T7 polymerase), and pGL3-Luc (encoding the firefly [FF] luciferase) were described previously (13, 31).

The DNA vaccine plasmids pCMV-Ub-N, pCMV-Ub-Gc, and pCMV-Ub-Gn (termed pN, pGc and pGn, respectively; CMV is cytomegalovirus) were generated using standard molecular cloning techniques. They contain the coding sequence of solely the nucleoprotein N (in pN), the glycoprotein Gc (in pGc), or the glycoprotein Gn (in pGn), each tethered to an N-terminal ubiquitin coding sequence. PCR was carried out with Phusion HotStartII enzyme (FinnZymes), and PCR products were cloned into the pCMV-Ub vector (16) using a BglII restriction site.

All ORFs derive from the CCHFV strain IbAr10200. The ORF of CCHFV nucleoprotein N was amplified from the pCAGGS_N plasmid (31) using the following primers: CCHF_N_BglII_F (*AGCCAGGAGATCT*GAAACAAGATCGAGGTG) and CCHF_N_BglII_R (*ATGCGCTGAGATCT*AATGATGTTAGCACTGGTGG). The ORFs of CCHFV glycoproteins Gn and Gc were amplified from the pCAGGS_GP plasmid (13) using the following primers: the pair CCHFV_Gn_Esp_F (*GCGTACCGTCTCAGATCT*TCAGAAGAACCCAGTGATGAC) and CCHFV_Gn_Esp_R (*GGTCTCCGTCTCAGATCT*TACAACCCAAGGAATTCTTTC) and the pair CCHFV_Gc_Esp_F (*GCCAACCGTCTCAGATCT*TTCCTAGATAGTACAGCTAAAGG) and CCHFV_Gc_Esp_R (*GGTCTCCGTCTCAGATCT*GCCAATGTGTGTTTTTGTAGAG), respectively. Roman letters are the sequences for CCHFV, and italicized letters are extra bases added for the cloning (containing restriction site and extra). Correct insertion was confirmed by DNA sequencing. Maxipreps of all DNA vaccine plasmids were prepared using an EZNA Endo-Free Plasmid DNA Maxi kit (Omega).

### Production, amplification, and purification of tc-VLPs expressing Renilla luciferase.

For production of tc-VLPs, we employed a slightly modified version of a previously described protocol (13). Briefly, HuH-7 cells grown in T 175-cm^2^ flasks in Dulbecco's modified Eagle's medium (DMEM) supplemented with 10% fetal calf serum (FCS), 2 mM l-glutamine, and 50 U/ml penicillin–50 μg/ml streptomycin were transfected with 11 μg of pCAGGS_V5_L_wt, 3.6 μg of pCAGGS_N, 3.6 μg of pT7riboSM2_vL_Ren, 9.1 μg of pCAGGS_GP, 9.1 μg of pCAGGS_T7, and 500 ng of a pGL3-Luc (firefly [FF] luciferase) control, using GeneJammer transfection reagent (Agilent Technologies). Four hours after transfection, the medium was replaced with fresh medium. Cell supernatants, designated passage 0, were collected at 72 h posttransfection and centrifuged at 12,000 × *g* for 5 min to remove cellular debris.

Amplification of tc-VLPs was conducted by transfer of the cell supernatants onto new HuH-7 cells that were pretransfected 20 h earlier with 11 μg of pCAGGS_V5_L_wt, 3.6 μg of pCAGGS_N, and 9.1 μg of pCAGGS_GP. Supernatants (passage 1) were collected 72 h later and amplified again to obtain tc-VLPs (passage 2). Briefly, HuH-7 cells pretransfected with plasmids encoding CCHFV L and N were infected with 10-fold dilutions of tc-VLP-containing supernatants and stained by immunofluorescence against the Renilla luciferase that is expressed by the VLPs. The titers were calculated on the basis of the dilution factors and the ratio between the numbers of cell nuclei and *Renilla*-positive cells. To assess tc-VLP amplification, REN-Luc and FF-Luc activities were measured in cell lysates at each passage and after polyethylene glycol (PEG) precipitation, using a Dual-Luciferase Reporter Assay System (Promega) and a Centro LB 960 microplate luminometer (Berthold Technologies).

### Immunization schedule.

Three immunizations were performed with a 4-week interval between the first and the second immunizations and then with a 3-week interval between the second and the third inoculations. Fifty micrograms of DNA plasmid/immunization/mouse (7 mice/group) was given via the intradermal route by Biojector immunization, followed by electroporation immediately after injection of DNA plasmids. VLPs were always injected intraperitoneally in 200 μl of saline at 10^6^ VLPs/mouse. The immunization schedule is presented in Fig. 1A. All mice were bled 2 days prior to the first immunization and 3 weeks after each immunization. All mice were bled after CCHFV challenge (at day of sacrifice). Serum was collected and kept frozen until analyzed.

IFNAR^−/−^ mice were divided into four groups (Fig. 1A). Two groups (named A and B, with 7 mice per group) received a combination of the CCHFV DNA plasmids pGc, pGn, and pN during primary and secondary immunizations. While group A received a further third dose of pGc, pGn, and pN, mice in group B were given CCHFV tc-VLPs i.p. as a third dose. Further, group C (6 mice) was given three immunizations with CCHFV VLPs, and group D (6 mice) received three immunizations with control plasmid DNA (pCMV-Ub).

### Detection of cytokines in serum.

Cytokine responses were analyzed using a Pro Mouse cytokine assay Th1/Th2 panel, according to the manufacturer's protocol (Bio-Rad, Richmond, CA). In brief, 50 μl of serum in assay diluent was added to cytokine beads labeled with anti-Th1 (IFN-γ, interleukin-2 [IL-2], and IL-12 p70) and Th2 (tumor necrosis factor-α [TNF-α], IL-4, IL-10, and granulocyte-macrophage colony-stimulating factor [GM-CSF]) cytokine antibodies. Standard curves for each cytokine were used to determine cytokine concentration/milliliter of serum.

### Challenge.

CCHFV strain IbAr10200 was propagated in SW13 cells. Viral titers were determined using standard methods (see above). At 6 weeks post-final immunization, all mice were challenged with 400 focus-forming units (FFU) of CCHFV i.p. (40 times higher than lowest lethal dose) and then monitored for up to 10 days with respect to clinical signs of disease, body weight, and survival. Due to the procedure protocol for animal experiments at our biosafety level 4 (BSL-4) facility, we could not follow the surviving mice longer that 10 days postchallenge.

### Viral RNA detection.

To inactivate virus, samples were treated with TRIzol LS reagent (Invitrogen, Carlsbad, CA, USA) and subsequently phase separated by chloroform (Merck, Billerica, MA, USA) treatment and centrifugation. Viral RNA was purified from the aqueous phase using a QIAamp Viral RNA minikit (Qiagen, Hilden, Germany) according to the manufacturer's instructions. The extracted RNA was analyzed by an S-segment-targeting real-time RT-PCR, as previously described (32). The same primers were included in an in-house version of a SYBR green-based assay using a SuperScript III Platinum SYBR green One-Step qRT-PCR kit (Invitrogen, Carlsbad, CA, USA). The cycling parameters were 20 min at 50°C, 5 min at 95°C, and 45 cycles of 10 s at 95°C, 10 s at 55°C, and 30 s at 68°C, followed by melting curve analysis with wavelength filter F1/F2 performed at the end of the assays. The cycling reactions were performed using a capillary Roche LightCycler 2.0 system. In addition, the primers were included in an endpoint RT-PCR using the SuperScript III One-Step RT-PCR System with a Platinum Taq DNA polymerase kit (Invitrogen, Carlsbad, CA, USA). The cycling conditions were 15 min at 50°C, 2 min at 94°C, 45 cycles of 15 s at 94°C, 15 s at 50°C, and 30 s at 63°C, followed by 5 min at 68°C, using a GeneAmp PCR System 2700 (Applied Biosystems, Foster City, CA, USA).

### Serology.

An inactivated CCHFV antigen was used for coating at a 1:300 dilution in 0.05 M Na_2_CO_3_-NaHCO_3_ buffer, pH 9.6, in flat-bottomed 96-well plates (PolySorp, Nunc, Thermo Scientific). The CCHFV antigen had been prepared by standard sucrose-acetone extraction from infected suckling mouse brain. CCHFV antigen was inactivated by gamma irradiation (dose, 30 kGy). Phosphate-buffered saline (PBS; pH 7.2) with 0.05% Tween 20 (Sigma-Aldrich) was used for blocking, as diluent, and as wash buffer. Mouse serum was titrated in 2-fold steps from a starting dilution of 1:50 in duplicate wells. A pool of known negative serum samples and a murine monoclonal antibody to CCHFV as a positive control were included on every plate. Horseradish peroxidase-conjugated polyclonal rabbit anti-mouse immunoglobulins (P0260; Dako) were used as tracers for the total Ig ELISA, and for the IgG subclass ELISAs peroxidase-conjugated goat anti-murine Fcγ 1 (catalog number 115-035-205), Fcγ 2a (115-035-206), Fcγ 2b (115-035-207), Fcγ 2c (115-035-208), and Fcγ 3 (115-035-209), all from Jackson ImmunoResearch, were used. An in-house substrate buffer (1 mM 3,5,3′,5′-tetramethylbenzidine in 0.1 M potassium citrate, pH 4.2, with 0.007% H_2_O_2_) was used for visualization of antibody binding. This reaction was stopped at a standardized time point with 2 M H_2_SO_4_, and the *A*_450_ was measured in an ELISA reader. For every titrated sample, *A*_450_ values were plotted against the sample dilution, and the equation for the linear part of the curve was determined by regression analysis. Antibody titers were then calculated as the dilution that would achieve an *A*_450_ value of 1. For total CCHFV Ig, a titer was determined for each individual mouse. In each subclass ELISA, two serum pools from two to four individuals having the highest and lowest total CCHFV Ig titers were titrated for each treatment group.

### B-cell epitope mapping.

A library of 288 CCHFV scan peptides (20-mers with an overlap of 10 amino acids) was printed on microarray slides for B-cell epitope mapping (33, 34), covering the sequence of the envelope glycoproteins of the Turkey strain (UniProt accession number C7F6X8) (peptides 1 to 168) and the N-capsid of the Turkey strain (UniProt accession number C7F6X7) (peptides 242 to 288). This library has been used and evaluated in a previous serology study with human CCHFV-positive sera (A. Fritzen, C. Risinger, A. C. Hitzeroth, N. Viljoen, F. Burt, N. Kjeldmand, G. Korukluoğlu, I. Christova, A. Mirazimi, and O. Blixt, unpublished data). Due to the fact that the Gc and Gn regions are known to be highly conserved (alignment between lbAr10200 [GenBank accession number Q8JSZ3, amino acids 516 to 1688] and the Turkey strain [UniProt accession number C7F6X8, amino acids 516 to 1688] shows an identity of 92% and a similarity of 96%), it was decided to perform the serology with the library at hand. Further epitopes might be identified in a future study with a library that is optimized for the lbAr10200 strain. In brief, serum was diluted 1:100 in PLI-P buffer (0.5 M NaCl, 3 mM KCl, 1.5 mM KH_2_PO_4_, 6.5 mM Na_2_HPO_4_, 3% BSA, pH 7.4). The subarray wells were filled with 300 μl of serum sample solution and incubated overnight (18 h) at room temperature on an orbital shaker. For the secondary staining, goat anti-mouse IgG-Cy5 (10 μg/ml; Sigma-Aldrich) was diluted 1:500 in PLI-P buffer, and 300 μl of this solution was used for incubation at room temperature (RT) for 1 h on an orbital shaker. All incubation steps were performed in a humidification chamber and were separated by three wash steps in PBS. After a final wash in PBS, slides were rinsed in water and dried by centrifugation. The slides were scanned with a ProScanArray microarray scanner. Spots were identified using automated spot finding with manual adjustments for occasional irregularities. The mean value of relative fluorescence intensity was used, and the spot intensities were determined by subtracting the median pixel intensity of the local background from the average pixel intensity within the spots. The results for five spots per peptide were averaged. To elucidate serum IgG responses against linear B-cell epitopes in surviving CCHFV-challenged mice, we selected the sera from group A and B mice at 10 days postchallenge for this study.

### Neutralization assay.

The titer of anti-CCHFV neutralizing activity in the serum was determined by microneutralization assay. Serum samples were heat inactivated for 30 min at 56°C. Serial 2-fold dilutions of serum were mixed with virus at a multiplicity of infection (MOI) of 0.05 (approximately 200 virus particles) and incubated at 37°C for 1 h before being added to Vero cells on a 96-well plate in triplicate. One hour later, medium was replaced with fresh DMEM supplemented with 2% FBS, 2 mM l-glutamine, and antibiotics (10 U/ml penicillin and 10 μg/ml streptomycin), and cell culture was continued for a further 24 h at 37°C. Cells were fixed with ice-cold acetone-methanol (1:1) for 30 min and stained for viral N for enumeration of fluorescent foci, as described previously (35). The total numbers of infected cells in positive controls and samples were counted, and the results were expressed as percent neutralization.
